# Coronary Artery Bypass Grafting Under ECPELLA Support After Out‐of‐Hospital Cardiac Arrest: A Case Report

**DOI:** 10.1002/ccr3.73000

**Published:** 2026-06-21

**Authors:** Hiroki Moriuchi, Kodai Yamada, Yusuke Tamaki, Toshiya Fukushima, Kohei Narayama, Takayuki Fujii, Nobuhiro Shimabukuro, Akihiko Yamauchi

**Affiliations:** ^1^ Department of Cardiovascular Surgery Yuuai Medical Center Okinawa Japan

**Keywords:** ECMO, Impella, OHCA, total arterial revascularization

## Abstract

The survival and social reintegration rate of out‐of‐hospital cardiac arrest (OHCA) remains extremely low. Venoarterial extracorporeal membrane oxygenation (VA ECMO) is used to support patients with cardiogenic shock. Combining Impella (Abiomed Inc., Danvers, MA, USA) with VA ECMO (ECPELLA) is a promising strategy to stabilize circulation and unload the left ventricle. We report a 46‐year‐old man who presented in cardiopulmonary arrest due to acute coronary syndrome. Coronary angiography showed multivessel disease, and echocardiography revealed an ejection fraction below 10%. ECPELLA was initiated immediately for hemodynamic stabilization and to promote myocardial recovery. After confirming neurological recovery, emergency coronary artery bypass grafting (CABG) using total arterial grafts and an aorta no‐touch strategy was performed under ECPELLA support. The postoperative course was uneventful, and all grafts remained patent. Cardiac function improved, and the patient returned to society 3 months after surgery. ECPELLA enables circulatory stabilization and myocardial recovery in patients with severe cardiogenic shock after cardiac arrest. Combining ECPELLA with total arterial revascularization may achieve excellent neurological and social outcomes, even in cases previously considered unsalvageable.

## Key Clinical Message

ECPELLA‐supported CABG can achieve social recovery in patients with out‐of‐hospital cardiac arrest.

**Abbreviations**
CABG: coronary artery bypass grafting
CPR: cardiopulmonary resuscitation
ECPELLA: combined support of ECMO and Impella
EF: ejection fraction
GEA: gastroepiploic artery
LAD: left anterior descending artery
LCx: left circumflex artery
LITA: left internal thoracic artery
LVDd: left ventricular end‐diastolic diameter
OHCA: out‐of‐hospital cardiac arrest
PDA: posterior descending artery
PL: posterolateral branch
RCA: right coronary artery
RITA: right internal thoracic artery
VA‐ECMO: venoarterial extracorporeal membrane oxygenation

## 1 Introduction

The survival and social reintegration rate of out‐of‐hospital cardiac arrest (OHCA) remains extremely low [1]. Venoarterial extracorporeal membrane oxygenation (VA ECMO) has been used to support patients with cardiogenic shock. However, the combination of Impella with VA‐ECMO (ECPELLA) has the potential to promote myocardial recovery.

This case is unique because the patient presented with OHCA, severe multivessel coronary artery disease, and extremely depressed left ventricular function. Despite these critical conditions, ECPELLA support allowed hemodynamic stabilization, left ventricular unloading, and assessment of neurological recovery before surgery. This case is also interesting because coronary artery bypass grafting (CABG) was performed with total arterial grafts and an aorta no‐touch strategy under ECPELLA support. This strategy may contribute to both acute recovery and favorable long‐term outcomes.

### 1.1 Case History

A 46‐year‐old man was transported to the emergency department in cardiopulmonary arrest. The patient had no prior medical history. The initial electrocardiogram showed ventricular tachycardia, and cardioversion and endotracheal intubation were performed in the ambulance during cardiopulmonary resuscitation (CPR). After the return of spontaneous circulation, the patient's hemodynamics remained unstable, so venoarterial extracorporeal membrane oxygenation (VA ECMO) was initiated through the right femoral artery and vein, and an emergency coronary angiography (CAG) was performed.

### 1.2 Investigations and Treatment

**FIGURE 1:**
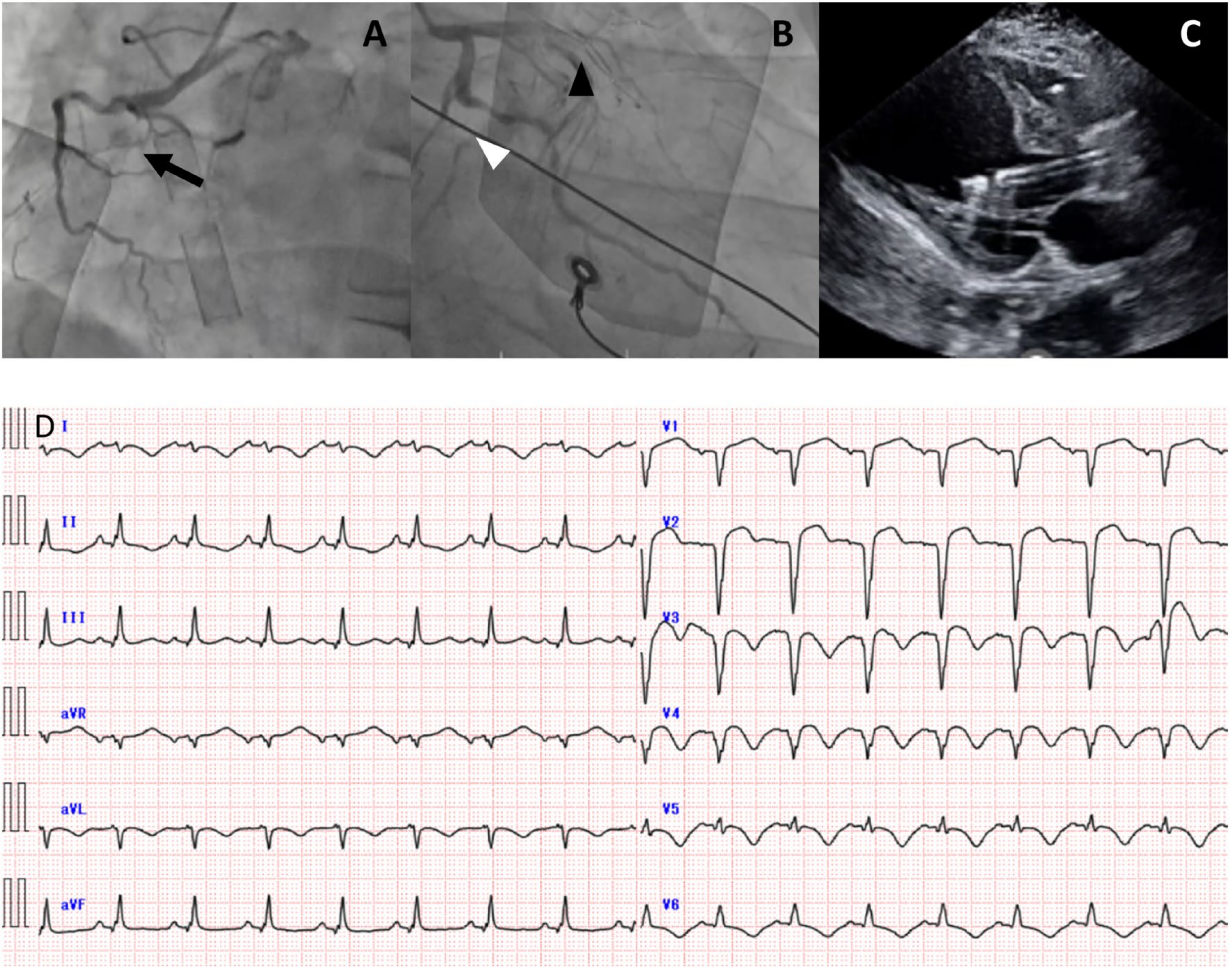
Coronary angiography showing occlusion of the proximal right coronary artery (A, black arrow) and the left anterior descending artery (B, black arrowhead), as well as severe stenosis of the left circumflex artery (B, white arrowhead). Echocardiography showed Impella positioned in a dilated left ventricle (C). Initial electrocardiogram showed ST changes in multiple lead (D).

**FIGURE 2:**
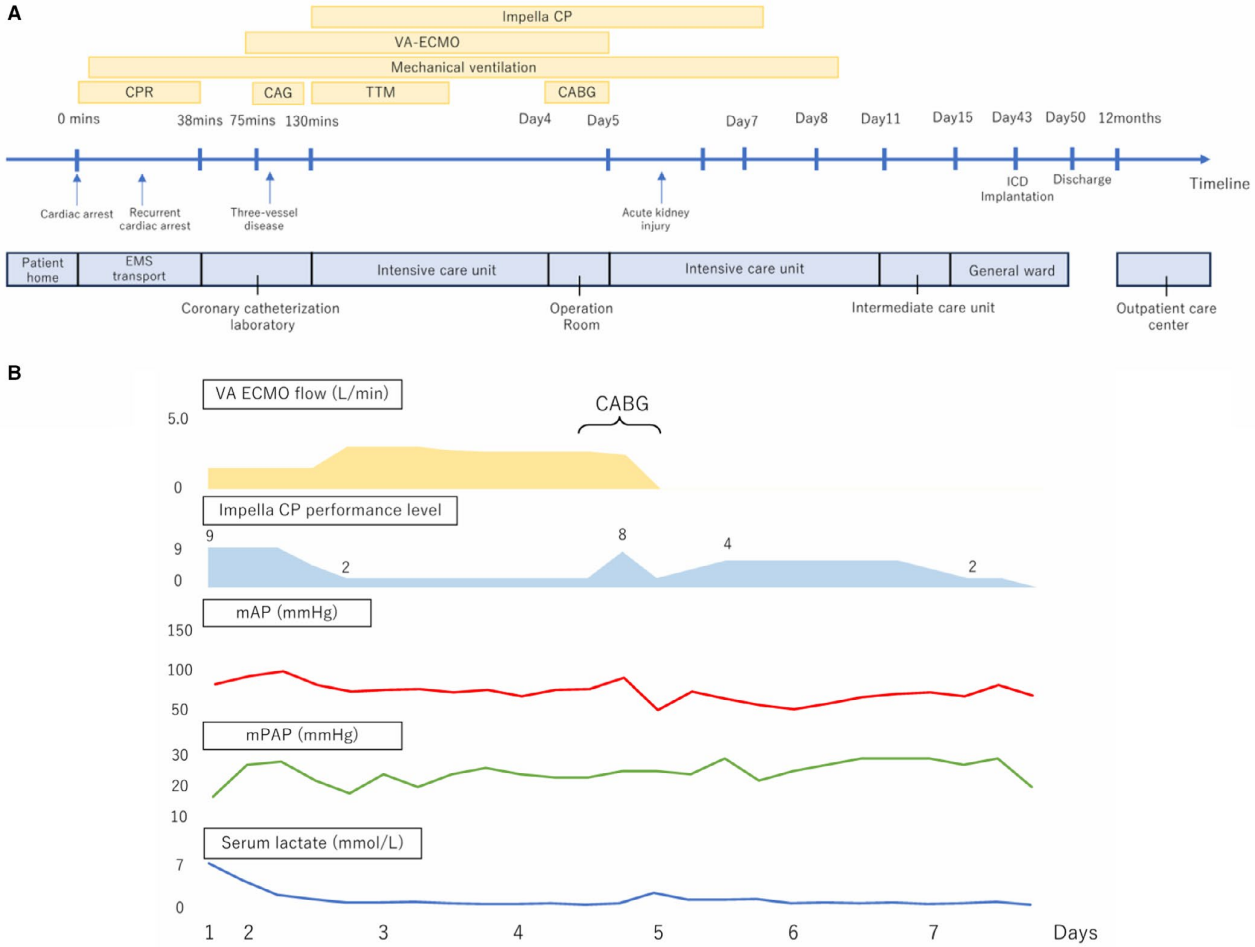
(A) The timeline of major events. (B) The hemodynamic status from onset to Impella removal. CAG, coronary angiography; CPR, cardiopulmonary resuscitation; EMS, emergence medical support; mAP, mean arterial pressure; mPAP, mean pulmonary arterial pressure; TTM, targeted temperature management.

CAG revealed multivessel coronary artery disease, including chronic total occlusion at the ostium of the right coronary artery (RCA) and the proximal portion of the left anterior descending artery (LAD), as well as 99% stenosis in the mid portion of the left circumflex artery (LCx). Collateral flow was observed from the septal branch to the posterior descending artery (PDA) and from the obtuse marginal branch (OM) to the posterolateral branch (PL) (Figure 1A,B). Echocardiography revealed severely depressed cardiac function, with a left ventricular end‐diastolic diameter (LVDd) of 65 mm and an ejection fraction (EF) of less than 10%. Therefore, an Impella CP device was inserted through the left femoral artery (Figure 1C). Figure 1D showed the electrocardiogram after ECPELLA insertion, demonstrating widespread ST‐segment changes in multiple leads. After confirming consciousness, emergency coronary artery bypass grafting (CABG) was performed. Anticoagulation therapy during ECPELLA support was performed with heparin, targeting an Activating clotting time of 200–250 s. Under general anesthesia with ECMO and Impella support, CABG × 4 was performed. The graft configuration was right internal thoracic artery (RITA) to the LAD, left internal thoracic artery (LITA) to the OM and PL branches, and right gastroepiploic artery (GEA) to the PD. During surgery, the ECMO flow was maintained at 2–4 L/min, and the Impella support level was set between P3 and P8. Even during heart displacement, careful volume management was performed to prevent inadequate Impella support due to left ventricular underfilling. Management was guided by transesophageal echocardiography and pulmonary artery catheter monitoring. Deep pericardial sutures were used to position the heart instead of a suction device. After completion of the anastomoses, the patient's hemodynamics improved, and the ECMO cannulas were removed. Figure 2A shows the timeline of major events, including a CPR duration of 38 min and 130 min from onset to Impella insertion.

### 1.3 Outcomes and Follow‐Up

**FIGURE 3:**
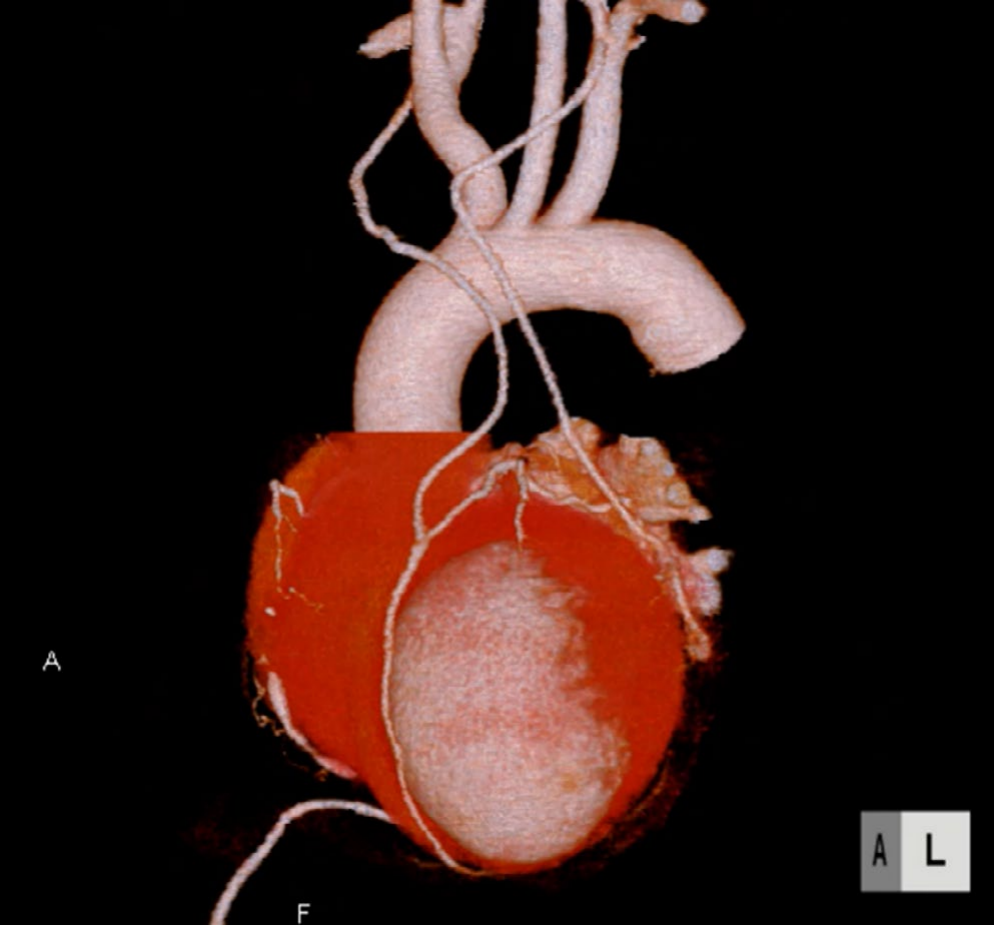
Postoperative computed tomography showing patency of all grafts.

The Impella was removed on POD 3. The patient was extubated on POD 4 and transferred from the intensive care unit on POD 7. Cardiac resynchronization therapy with defibrillator implantation was performed on POD 30, and he was discharged ambulatory on POD 37. Figure 2B and Table 1 shows the hemodynamic status from onset to Impella removal. Postoperative CT revealed patency of all grafts, and echocardiography demonstrated improved cardiac function with favorable reverse remodeling, with EF 42% and LVDd 52 mm (Figure 3). The patient achieved good neurological recovery and returned to society 3 months after surgery.

**TABLE 1:** Hemodynamic parameters during ECPELLA support.

Parameter	On arrival	Day 2				Day 3				Day 4 (CABG and ECMO removal)				Day 5				Day 6				Day 7 (impella removal)			
Time (h)	18	0	6	12	18	0	6	12	18	0	6	12	18	0	6	12	18	0	6	12	18	0	6	12	18
mPAP (mmHg)	17	27	28	22	18	24	20	24	26	24	23	23	25	25	24	29	22	25	27	29	29	29	27	29	20
MAP (mmHg)	80	90	96	79	71	73	74	70	73	65	73	74	88	48	71	62	54	49	56	64	68	70	65	79	66
PaO_2_ (mmHg)	112	169	126	178	193	79	85	80	76	72	96	73	194	109	128	86	96	90	82	112	107	86	96	68	109
Lactate (mmol/L)	6.6	4.1	2	1.4	0.9	0.9	1	0.8	0.7	0.7	0.8	0.6	0.8	2.3	1.3	1.3	1.4	0.8	0.9	0.8	0.9	0.7	0.8	1	0.6
Impella CP (P‐level)	9	9	9	5	2	2	2	2	2	2	2	2	8	2	4	6	6	6	6	6	6	4	2	2	—
ECMO flow (L/min)	1.85	1.85	1.85	1.85	3.75	3.75	3.75	3.4	3.3	3.3	3.3	3.3	3	—	—	—	—	—	—	—	—	—	—	—	—

Abbreviations: CABG, coronary artery bypass grafting; ECMO, extracorporeal membrane oxygenation; ECPELLA, combined support with VA‐ECMO and Impella; MAP, mean arterial pressure; mPAP, mean pulmonary arterial pressure; PaO_2_, arterial oxygen pressure.

## 2 Discussion

Acute coronary syndrome is one of the most common causes of cardiac arrest. Out‐of‐hospital cardiac arrest (OHCA) is associated with high mortality, and the rate of social reintegration remains very low, at approximately 3.3% [1]. VA ECMO has been used to treat cardiogenic shock, however, recently, the combined use of Impella has been associated with improved outcomes due to effective LV unloading [2, 3, 4]. Previous studies have reported that the perioperative use of VA‐ECMO combined with Impella (ECPELLA) is associated with better cardiac surgical outcomes compared with VA‐ECMO alone [5]. VA ECMO can provide systemic perfusion but increases LV pressure, which may impair myocardial recovery. In contrast, Impella unloads the left ventricle and thereby reduces myocardial oxygen consumption. ECPELLA combines these advantages by providing both systemic perfusion and active LV unloading, which may stabilize hemodynamics and organ perfusion before surgery, optimize perioperative management, and facilitate myocardial recovery.

In this case, the patient was transported to the hospital in cardiopulmonary arrest, and VA ECMO was initiated emergently. CAG revealed severe coronary artery disease and extremely low EF, prompting Impella insertion to provide hemodynamic support and LV unloading. After confirming neurological recovery and achieving stable hemodynamics and organ perfusion, CABG was performed. Given the patient's young age, very complex coronary anatomy, and severely depressed cardiac function, CABG with total arterial grafting was chosen. Total arterial revascularization using bilateral ITA has been associated with superior long term graft patency and improved survival compared with vein grafts, particularly in younger patients with low EF, as in this case [6, 7]. In addition, an aorta no‐touch strategy was adopted, which is considered effective during CABG in patients supported with Impella.

During ECPELLA supported CABG, careful hemodynamic management is essential. Bleeding, fluid administration, and cardiac positioning change left ventricular volume. These changes can cause device alarms and hemodynamic instability. Therefore, close communication with the anesthesiologist and perfusionist is very important. Transesophageal echocardiography must be used to assess left ventricular volume. Venous drainage and Impella support level should be adjusted to maintain a stable left ventricular volume [3, 8, 9]. Cardiopulmonary bypass was prepared as a backup option in case of massive bleeding or insufficient support with VA‐ECMO.

During ECPELLA support, major complications such as bleeding, limb ischemia, and embolism can occur and may lead to fatal outcomes [2]. Therefore, optimal anticoagulation management and timely device weaning are crucial. Fortunately, no major complications occurred in this case. Nersesian et al. reported that CPR before Impella insertion and a serum lactate level > 8 mmol/L are associated with 30‐day mortality. However, our patient fully recovered with appropriate perioperative management [10].

ECPELLA allowed hemodynamic stabilization and effectively improved systemic circulation while unloading the left ventricle. Rapid initiation of ECPELLA support and timely surgery contributed to favorable neurological recovery and social reintegration, which are uncommon in such severe cases. Further studies are needed to optimize ECPELLA management strategies.

## Author Contributions

**Hiroki Moriuchi:** conceptualization, writing – original draft. **Kodai Yamada:** investigation. **Yusuke Tamaki:** conceptualization. **Toshiya Fukushima:** conceptualization. **Kohei Narayama:** conceptualization. **Takayuki Fujii:** conceptualization. **Nobuhiro Shimabukuro:** conceptualization. **Akihiko Yamauchi:** conceptualization.

## Funding

The authors have nothing to report.

## Ethics Statement

Written informed consent was obtained from the patient and their family.

## Conflicts of Interest

The authors declare no conflicts of interest.

## Data Availability

All relevant data are within the manuscript.
